# Long term functional outcome and quality of life of patients with refractory juvenile idiopathic arthritis treated with etanercept: results of the dutch arthritis and biologicals in children register

**DOI:** 10.1186/1546-0096-12-S1-P28

**Published:** 2014-09-17

**Authors:** Janneke Anink, Femke HM Prince, Maryanne Dijkstra, Marieke H Otten, Marinka Twilt, Rebecca ten Cate, Simone L Gorter, Yvonne Koopman-Keemink, Marion AJ van Rossum, Esther PA Hoppenreijs, Lisette WA van Suijlekom-Smit

**Affiliations:** 1 Paediatric Rheumatology, Erasmus MC Sophia Children's Hospital, Rotterdam, Netherlands; 2 Paediatric Rheumatology, LUMC, Leiden, Netherlands; 3 Rheumatology, MUMC, Maastricht, Netherlands; 4 Paediatric Rheumatology, Hagaziekenhuis Juliana Children's Hospital, The Hague, Netherlands; 5 Paediatric Rheumatology, Emma Children's Hospital AMC and Reade , Amsterdam, Netherlands; 6 Paediatric Rheumatology, Radboud MC and St. Maartenskliniek, Nijmegen, Netherlands

## Introduction

Juvenile idiopathic arthritis patients refractory to methotrexate are eligible for treatment with biologic agents. A longitudinal sub-analysis (n=53) of the Dutch Arthritis and Biologicals in Children register previously showed that disability and health related quality of life (HRQoL) improved shortly after treatment with etanercept.

## Objectives

To investigate long term functional outcome, HRQoL and treatment changes in the same patients, who started etanercept >5 years ago.

## Methods

Patients were traced and questioned on education and employment. Data on recent disease status, comorbidities and structural damage were retrieved. Disability was assessed by (Child) Health Assessment Questionnaire ((C)HAQ), HRQoL was measured by Child Health Questionnaire, Short Form 36 and Health Utilities Index Mark 3. Linear mixed models were used to analyze changes over time.

## Results

43 patients (81% response) started etanercept median 8.5 years (IQR: 7.7-10.3) ago. Median age was 22 years (IQR: 18-24). 42% had a (C)HAQ of 0.00. HRQoL was similar to HRQoL shortly after start of etanercept, except for the domains bodily pain and general health perception, which deteriorated to levels comparable to those at start of etanercept. VAS pain also worsened (median 12 (IQR:2-43)), however pain indicated by VAS remained lower than at start of etanercept. The unemployment rate (12%) was comparable to the general population; educational level was higher (77% of patients >17 years had achieved at least upper secondary education). 40% of patients switched to other biologic agents, 40% were still using etanercept and 20% were off anti-rheumatic treatment. 14% had had joint surgery. There were no reports of malignancies.

## Conclusion

The improvement in HRQoL after start of etanercept was sustained after 8.5 years. Disability was low. On many aspects of daily life, patients functioned comparably to or better than the general population. The need for surgery for 14% of patients stresses the importance of early treatment of JIA. Chronic pain - also when the disease is inactive - remains an important issue that should not be overlooked.

## Disclosure of interest

J. Anink: None declared., F. H. Prince: None declared., M. Dijkstra: None declared., M. H. Otten: None declared., M. Twilt: None declared., R. ten Cate Grant / Research Support from: Pfizer (outside submitted work), Consultant for: Pfizer, S. L. Gorter: None declared., Y. Koopman-Keemink: None declared., M. A. van Rossum: None declared., E. P. Hoppenreijs: None declared., L. W. van Suijlekom-Smit Grant / Research Support from: Dutch Board of Health Insurances, Pfizer, AbbVie, Consultant for: Pfizer, Novartis, Roche Bristol-Meyers Squibb.

